# Stricture of the Rectum

**Published:** 1886-12

**Authors:** A. E. Hoadley

**Affiliations:** Professsor of Anatomy, Chicago College of Physicians and Surgeons, Professor of Surgery, Chicago Policlinic; 683 Washington Boulevard


### A REPORT OF FIVE CASES OF STRICTURE OF THE RECTUM, WITH REMARKS ON THE TREATMENT OF ITS MORE SEVERE FORMS.

*By* A. E. HOADLEY, M.D., *Professsor of Anatomy, Chicago College of Physicians and Surgeons, Professor of Surgery, Chicago Policlinic.*

[Read before the Chicago Medical Society, November 1st.]

For the purpose of demonstrating that one plan is better than another, in the surgical treatment of any disease, it is necessary, in a measure at least, to show the results of both methods.

With this object in view, I have selected the following five cases, some of which in themselves illustrate more than one plan of treatment. Such cases must necessarily show some errors of judgment, or they could not serve the double purpose of illustrating good and bad treatment. Furthermore, a badly treated case honestly reported often teaches a better lesson than the report of one that has been skillfully managed.

CASE I.—Mr. J. H. German, engineer, forty years of age, gave a history of hæmorrhoids of eight years' standing, and stricture of two years. Examination revealed a hard carcinoma of the rectum within one and a half inches of the anus, and immovable on account of adhesions to the sacrum. All of the adjacent parts were infiltrated, and the bowel completely occluded. The general condition of the patient was very bad. He was emaciated, bed-ridden, and suffered almost constant pain. His abdomen was much swollen and very tender, the bowels not having moved for a period of two weeks. All food was immediately vomited. He only took small quantities of wine and water. Pulse was 120 and feeble. His temperature, 102° F. At the first examination I succeeded with the finger in changing somewhat the relation of the cancerous mass that protruded into the rectum from the front, and which was also adherent to the posterior wall. The adhesions being very carefully separated, the tumor could be brought down to a slight extent, which enabled me to pass the nozzle of a syringe beyond the stricture. This was done without any laceration of the tissues of the tumor, a thing always to be guarded against in the examination of malignant stricture of the rectum. With the nozzle of the syringe in position I injected a half pint of soap-water which immediately escaped and brought with it a little fecal matter and considerable gas. The injection was ordered to be repeated through the afternoon and evening as often as the patient could endure it. On the following day I was informed that five injections had been given, with the effect of bringing away considerable fecal matter and gas, resulting in great relief to the patient. He could then take beef-tea and retain it. His pulse was 100. His temperature 100° F. As an attempt at opening the rectum was so painful, he was allowed to go three days without further treatment, when it again became necessary to do something for him. Ether was administered and, by careful use of the finger, sharp curette, and knife, an opening large enough to admit a finger was formed. This operation was followed by great relief generally, but there remained severe tenesmus and pain with and after each act of defecation. Two other like operations were performed, which resulted in the formation of a fair canal through the cancerous mass, but without relief from pain. The patient had from the first refused to have anything like a surgical operation performed. What he had already endured, in the way of operations, was regarded by him as a means of stretching his stricture. But the patient was getting discouraged, and had decided to die without further treatment, when, with great difficulty, I induced him to submit to one more stretching operation. At this time I divided the lower end of the growth and the sphincter ani with one stroke of the knife. There was but little hæmorrhage, which ceased entirely within five minutes. With the exception of the occurrence of a small and very painful ischio-rectal abscess, which developed after this last operation, he suffered no more pain in the ano-rectal region. There was more or less pain in the lower part of the abdomen and back, necessitating the use of morphine once or twice a day. The relief obtained by *the division* of the sphincter was ten-fold greater than that from all the other operations together. A persistent diarrhœa, from extension of the disease, hastened his death, which occurred two months later. Although there was rectal incontinence, he would have sufficient warning to prepare himself for the coming passage. What I most regret in the treatment of this case is, that I did not, at the first operation, divide the sphincter and the cancer above it, so that there would have been perfect freedom for the discharges, and rest for the sphincter. Had I done this my patient would probably have had one month more of comparative freedom from pain, and would not have suffered the pains of an ischio-rectal abscess.

CASE II. Mr. G. English, a laborer, 42 years old, gave a history of hæmorrhoids and stricture of five years' standing, and a very unsatisfactory history of syphilis. Examination revealed, within two inches of the anus, a firm, unyielding stricture, which barely admitted the tip of my index finger. The most distressing conditions were diarrhœa and tenesmus. With some stretching and nicking, I was soon able to pass my finger beyond the first joint through the stricture. As syphilis could not be excluded, I put him on large and increasing doses of iodide of potassium, and commenced systematic dilatation, passing a bougie twice a week for four weeks, at which time I could, without difficulty, pass a number 10 rectal bougie one inch in diameter. There was no particular irritation at the seat of the stricture. He could, after that, defecate without difficulty. Further dilatation was stopped, and the iodide of potassium continued in forty-grain doses three times a day. Two months made no change in the quality or quantity of the stricture-tissue. The passage was still so large that there was no obstruction, but the bowels were very irritable, the pain and diarrhœa gradually getting worse, and the patient evidently losing flesh. He begged to have something done for his relief, and readily consented to have the stricture and sphincters divided, which was thoroughly done, and the wound packed with gauze on which a little dry persulphate of iron had been sprinkled. Great relief followed the operation, and the diarrhœa immediately subsided. There was no pain, his appetite returned, and he slept well, until the fourth day, when he suddenly imagined that he felt very bad because he had had no movement of his bowels in the meantime. A small dose of licorice powder at night relieved his bowels, and also his mind. The following two months he was annoyed by incontinence of gas and liquid fæces, but he stated that the annoyance of incontinence was nothing compared to the distress caused by the stricture before its division. An examination two months after operation showed that the incision at the stricture and in the bowel below, including half of the internal sphincter, was perfectly healed. The wound of the external spincter and integument was nearly closed, with good prospect of its being entirely healed within another month. At the site of the stricture there is nothing to indicate that it ever existed. The bowel, on a superficial examination, appears soft and pliant, and as capacious as normal, but with care one can discover that it is slightly thicker at this point than above or below. The thickened condition extends up and down for about one inch and a half. It is soft, and feels like a slight swelling of the mucous membrane. His general condition is improving every day, and he feels much better than at any time during the past year.

CASE III.—Mrs. K., American, aged thirty-nine years, mother of three children, gave a history of stricture of rectum of nine years' standing. She had been treated by several physicians in various ways, with but little benefit. I first saw the case on July 1st, 1886. She was a pitiable object; emaciated to a remarkable degree; walked with great difficulty; had from fifteen to twenty movements of the bowels every day, and every passage was attended with burning, tenesmic pain. On examination I found an almost impervious stricture within one inch of the anus. Vaginal examination showed that there was much mischief above the stricture. I informed her that an immediate operation was necessary to save her life. Still one week went by before she was admitted to the Presbyterian Hospital, in such a deplorable condition that I dreaded to operate lest she die on the table.

I first divided the stricture so that I could introduce my finger, when somewhat to my surprise I found that the rectum contained a carcinomatous growth almost occluding the canal, higher than I could reach with my fingers. It was necessary to divide the tissues posteriorly to admit my fingers, for fear of lacerating the tumor through into the peritoneum. After working my finger as far up the rectum as possible, I divided the sphincters and tissues back to the coccyx, when I was able to reach still higher, but could not reach the top of the mass, which was quite firmly adherent in all directions. I next made a deep incision through it posteriorly nearly to the sacrum, from a point as high up as I could reach with my finger down to the incisions already made through the external tissues. I then quickly placed a piece of gauze in the wound with pressure upon it, to try to prevent bleeding. Although the hæmorrhage was not very free, it was necessary to save every drop of blood possible, as my patient was *in ex-*

*tremis.* With some difficulty I succeeded in getting a large-sized drainage-tube above the stricture, for the purpose of relieving the bowels of gas and liquid feces. I then finished packing the rectum, leaving the tube in, and, after securing all with a T bandage, put the patient to bed. For four days she lingered at death's door, and then quickly rallied and was removed from the hospital in a week from the time of the operation. In two weeks she was walking about. Her general condition was improving rapidly; the diarrhœa had stopped, and her appetite was good. At the end of five weeks she left the city on a visit, where, at last accounts, she still was. Three months after the operation, she was feeling very comfortable and had gained six pounds in weight, and said in a letter to her mother, " if Dr. H. could see me now he would not think that I had a cancer." I heard from her one week ago, when she was still comfortable, and able to walk out every day.

The following case will illustrate some of the dangers attending the practice of forcibly dilating malignant strictures.

CASE IV.—A German lady, fifty-six years old, mother of several children, had always enjoyed good health until the last three years, when she became aware of the presence of a stricture of the rectum. She had done little for it until it had so nearly closed the bowel that she could endure the distress no longer, when she applied for treatment. Examination disclosed a close, firm, annular stricture, within one inch of the anus. The stricture with the sphincters were divided, and on introducing the finger, the bowel was found blocked up with other strictures, undoubtedly malignant, and of irregular formation, as far as the finger could reach. These were dilated with the fingers, the tissues being quite friable and yielding readily under pressure. The canal was opened as far as I

could reach, without lacerating the rectum itself. Violent inflammation supervened, involving the pelvic cellular tissue, extending up into the abdomen, and for five days it endangered the life of the patient. She made a very slow and tedious recovery, stating that she believed she was made worse by the operation. She went away, and I have heard nothing from her since. As to this case, it is my conviction, that had I incised those strictures, instead of stretching them, my patient would have been spared the dangerous ordeal of an inflammation and would have derived benefit and comfort from the operation.

CASE V.—An American lady, sixty-seven years old, well preserved and remarkably healthy until five years ago, when she became aware that she was afflicted with stricture of the rectum. She could give no history of any cause for it. She had had four children ; her labours had been normal and easy, and from every one of which she made a rapid and perfect recovery. Two years ago she had an ischio-rectal abscess, which resulted in the formation of a fistula, but which did not prove troublesome until some four or five months prior to my first visit, when she again suffered from another abscess and then from a general suppurating inflammation of the whole ano-rectal region. At this time Dr. G. E. Brinkerhoff was called to attend her, and recognizing the necessity of operative treatment he called me in consultation. Under the influence of an anæsthetic, an examination was made, which revealed two or three open sinuses and as many fluctuating abscesses in the ischio-rectal region, with a very firm annular stricture of the rectum within one and a half inches of the anus ; also, a carcinoma of the os uteri and upper part of the vagina. The vaginal walls were agglutinated below, but were easily separated, making the diagnosis easy and certain. It would have been better for the patient if the cancer had not been discovered. She was weak, emaciated and cachectic, which seemed as much due to the cancer as to the bowel trouble, and there appeared to be little that could be done for her. I therefore opened the abscesses and sinuses freely, packing them with iodoform-gauze, and divided the stricture without dividing the sphincter, contenting myself with dilating it. The operation was for the relief of pain, as it was believed that the patient had but a short time to live. The relief following the operation was great. The inflammation subsided, her bowels moved easily, and her general condition improved. The cancerous cachexia disappeared and she began to believe that she would get well, when another abscess made its appearance, causing great suffering and prostration. The attending physician wished to open this for her, but she refused and suffered it to break, which it did within the bowel, giving some relief; but as the abscess cavity continued to refill she was not fully relieved until some two weeks later, when the skin covering the sinus gave way, making a complete fistula, with relief of all pain. This condition did not last very long, for the stricture again became troublesome, and in making an examination, which I did with the consent of Dr. Brinkerhoff, I found that the stricture was as closely contracted as at the time of the operation, three and one-half months before. The cancer is about the same and gives her no trouble. Her general condition is comparatively good, and there is no evidence of immediate dissolution. With the present condition of the stricture, however, it is probable that she will soon be prostrated again. With these facts before her she declines another operation. In this case, again, I have to regret that I did not divide the sphincter with the stricture down to the cellular tissue beneath. By doing this I could have opened the fistulous tract throughout, and the stricture could not have again united, so soon at least, and the patient's last days would have been more comfortable. As it is, she may continue to suffer to the end of her life.

From a study of the foregoing cases we may deduce the following principles, which are in accord with the teachings of the best surgeons of the day.

*First.*—It is dangerous to practice divulsion of malignant stricture of the rectum.

*Second.*—Division of a severe stricture of the rectum without dividing the sphincters is of little practical value.

*Third.*—Division of malignant strictures, with the sphincter, gives great relief and tends to prolong life.

*Fourth.*—Division of severe non-malignant stricture, with the sphincters, gives great relief and tends to perfect cure.

*Fifth.*—Division of strictures, whether malignant or not, and of the sphincters, is attended with little danger to life. All severe strictures should be treated by complete division, the cut including all the tissues below and back, as far as the tip of the coccyx. This operation affords, in cases of malignant stricture, the greatest possible measure of relief, and in non-malignant stricture the best means of permanent cure at our command.

683 Washington Boulevard.

